# Standard versus double dosing of beta-lactam antibiotics in critically ill patients with sepsis: The BULLSEYE study protocol for a multicenter randomized controlled trial

**DOI:** 10.1186/s12879-025-10747-3

**Published:** 2025-03-21

**Authors:** M. M. B. Horstink, D. R. Geel, C. A. den Uil, P. E. Deetman, H. Endeman, A. Abdulla, T. M. Bosch, W. J. R. Rietdijk, F. W. Thielen, J. J. Haringman, P. van Vliet, T. A. Rijpstra, C. Bethlehem, A. Beishuizen, A. E. Muller, B. C. P. Koch

**Affiliations:** 1 Department of Intensive Care, Maasstad Hospital, Rotterdam, The Netherlands; 2 Rotterdam Clinical Pharmacometrics Group, Erasmus MC, Rotterdam, The Netherlands; 3 Department of Hospital Pharmacy, Erasmus MC, Rotterdam, the Netherlands; 4 Department of Intensive Care, Albert Schweitzer Hospital, Dordrecht, The Netherlands; 5 Department of Intensive Care, OLVG, Amsterdam, The Netherlands; 6 Department of Intensive Care, Erasmus MC, Rotterdam, The Netherlands; 7 Department of Clinical Pharmacology & Toxicology Maasstadlab, Maasstad Hospital, Rotterdam, The Netherlands; 8 Department of Hospital Pharmacy, Maasstad Hospital, Rotterdam, The Netherlands; 9 School of Health Policy & Management, Erasmus University, Erasmus Centre for Health Economics Rotterdam, Rotterdam, The Netherlands; 10 Department of Intensive Care, Isala Hospital, Zwolle, The Netherlands; 11 Department of Intensive Care Haaglanden Medical Center, The Hague, The Netherlands; 12 Department of Intensive Care, Amphia, Breda, The Netherlands; 13 Department of Intensive Care, Frisius MC, Leeuwarden, The Netherlands; 14 Department of Intensive Care, Medisch Spectrum Twente, Enschede, The Netherlands; 15 Department of Medical Microbiology, Haaglanden Medical Center, The Hague, The Netherlands

**Keywords:** Sepsis, Beta-lactam, Antibiotics, Critically ill, Intensive care, Randomized controlled trial, Mortality, Cost-effectiveness analysis

## Abstract

**Background:**

Sepsis and septic shock are significant global healthcare challenges with high mortality rates. Effective management requires timely and adequate antimicrobial therapy. Beta-lactam antibiotics, commonly used in patients with sepsis, are crucial for treating these infections. However, standard dosing often leads to insufficient plasma levels due to dynamic physiological changes in critically ill patients.

Previous randomized controlled trials highlighted the need for timely dose adjustments to improve clinical outcomes. This is the study protocol for the BULLSEYE trial in which we aim to optimize antibiotic treatment during the initial 48 h of sepsis by comparing standard to double dosing of beta-lactam antibiotics.

**Methods:**

This open-label, multicenter, randomized controlled trial will compare standard to double dosing of beta-lactam antibiotics (cefuroxime, ceftazidime, ceftriaxone, cefotaxime, amoxicillin, amoxicillin/clavulanic acid, flucloxacillin, meropenem, and piperacillin/clavulanic acid) in critically ill patients with septic shock. Participants will be randomized into two arms: the control arm receiving standard care, and the intervention arm receiving double antibiotic doses for 48 h, irrespective of renal function. Following this period, all patients will receive standard doses as per local protocol. The primary outcome is all cause 28-day mortality, with secondary outcomes including 90-day, 365-day, hospital and ICU mortality, hospital and ICU length of stay, SOFA scores, time to shock reversal, microbiological eradication, clinical cure, pharmacodynamic target attainment, safety, quality of life, and medical consumption.

**Discussion:**

The BULLSEYE trial aims to improve sepsis treatment in critically ill patients. Despite anticipated recruitment challenges, its large sample size ensures robust comparability. This pivotal trial could significantly impact sepsis treatment, leading to better clinical outcomes.

**Trial registration:**

EU_CT 2024–512950-13–00. Protocol version 2.3, protocol date 09–12-2024. Prospectively registered on 09–01-2025 at Clinicaltrails.gov nr. NCT06766461.

## Background

Sepsis and septic shock represent significant global healthcare challenges, annually affecting millions worldwide and ranking among the leading causes of mortality in hospitalized patients. In the Netherlands, severe sepsis accounts for approximately 0.6% of hospital admissions and 11% of intensive care unit (ICU) admissions, translating to an estimated 8,000–9,000 ICU admissions annually with a median length of stay of 13.3 days [1]. Mortality rates range from 20 to 54%, underscoring the critical nature of effective management strategies [2–6]. Furthermore sepsis and septic shock can cause long-term or lifelong disabilities such as Post Sepsis or Post Intensive Care syndrome due to the impact of ICU admission, medical condition and treatment [7, 8]. The key to improving outcomes for severe infections, such as sepsis and septic shock, lies in the timely and adequate administration of antimicrobial therapy.

Beta-lactam antibiotics are amongst the most commonly used antibiotics to treat sepsis. Their antimicrobial efficacy is determined using the pharmacodynamic target (PDT). The PDT is defined as the unbound antibiotic concentration (f) above the minimal inhibitory concentration (MIC): the lowest concentration needed to prevent bacterial growth. In beta-lactam antibiotics the PDT in critically ill patients is described as 100%fT > MIC (or more aggressively 100%fT > 4xMIC) [9], meaning that the unbound concentration stays above the MIC for 100% of the time (T).

During the first hours of sepsis and associated resuscitation, rapid dynamic changes in physiology occur, including augmented clearance, renal or hepatic dysfunction, changes in albumin and increased volume of distribution [10]. In this phase patients show significant interindividual variability in pharmacokinetic parameters, with a more than twofold variation of both volume of distribution and drug clearance [11]. Consequently, standard antibiotic dosing, as established in non-critically ill, appears to be insufficient in this population. Previous research by our group demonstrated that 40% of ICU admitted sepsis patients does not reach 100%fT > MIC and even more than 75% of the patients does not reach 100%fT > 4xMIC [12]. These findings are in line with other studies [13, 14].

To optimize dosing, the DOLPHIN study was carried out [14, 15]. This study aimed to improve sepsis treatment with Model-Informed Precision Dosing, using Therapeutic Drug Monitoring (TDM) in combination with PK modeling software. One of the limitations of the DOLPHIN study was, that, due to the study design and laboratory TDM availability, results and dose adjustments were available only 36–48 h after antibiotic initiation [16]. Consequently, standard dose had been administered to all patients in anticipation of dosing advice. Considering the “golden hour of sepsis”, and the importance to treat as soon and as good as possible, this time window is too long. A post-hoc analysis from the DOLPHIN study confirmed this: patients whose doses were adjusted based on TDM within 24 h after treatment initiation, had better clinical outcomes (amongst all: shorter ICU stay) compared to those receiving standard dosing [17]. Individual predictions using Model-Informed Precision dosing tools showed that doubling the dose would result in adequate target attainment in these patients [14], especially in the first 48 h of treatment. This is in concordance with other proposed dosing regimens in literature [18–20].

Therefore, this trial aims to investigate the effect of double dosing of beta-lactam antibiotics during the initial 48 h of septic shock on all cause 28-day mortality in critically ill patients.

## Methods and design

### Design

This is an open label, multicenter, randomized controlled trial, conducted in the Netherlands. At the start of the study period, participants will be randomized into two study arms. The control arm will receive standard care. The intervention arm will receive a double starting dose of antibiotics upon admission and will continue this double dose for 48 h. After 48 h all patients will receive the standard dose according to local protocol. Data collection will continue for a total duration of 12 months, including carrying out questionnaires regarding health-related quality of life and medical consumption at 3 and 12 months after inclusion.

### Participants

Participants include adult patients with septic shock, admitted to the ICU. Standard treatment must include, but is not limited to, beta-lactam antibiotics.

### Inclusion criteria

To be eligible to participate in this study, a subject must meet all of the following criteria:

-  ≥ 18 years of age
- Receiving intravenous antibiotic therapy of the target drugs (either intermittent or continuous infusion of beta-lactam antibiotics, depending on the local protocol)
- Primary infection
- Admitted to the ICU
- Meeting the Sepsis-3 criteria for septic shock: sepsis in addition to shock requiring the start of vasopressors to maintain a mean arterial pressure 65 mmHg or greater, and a serum lactate level greater than 2.0 mmol/L following “adequate fluid resuscitation” [21]

### Exclusion criteria

A potential subject who meets any of the following criteria will be excluded from participation in this study:

- Patient or legal representative not available to give informed consent within 72 h after admittance
- Pregnancy
- Admittance for burn wounds
- Patients receiving target antibiotics only as prophylaxis within the context of Selective Digestive tract Decontamination (SDD)
- Enrolment in another interventional trial
- A patient who received the study antibiotic for more than 24 h before inclusion
- A patient receiving extracorporeal membrane oxygenation (ECMO)
- A patient who is already treated with a double dose of antibiotics based on suspected infection

### Sample size calculation

The sample size calculation was based on the available mortality data from the DOLPHIN trial [14]. It was hypothesized that 28-day mortality will decrease from 28 to 20%. Furthermore, it was assumed to have 80% power, 5% two-sided alpha level and 5% loss-to-follow up. Therefore, the final sample requires 988 patients (494 patients in each treatment arm). The power calculation was performed using G*Power. The 8% mortality decrease is clinically meaningful and is realistic in early intervention sepsis studies [22].

### Study procedures and data collection

#### Screening procedure

Screening will take place at the participating hospital sites by the treating physician. If possible informed consent will be obtained before inclusion. If this is not possible due to the medical condition of the patient, the patient will be included and deferred consent will be obtained within 72 h from the patient or their legal representative.

#### Randomization, blinding and treatment allocation

This is an open label study where patients will be randomly assigned in a 1:1 allocation ratio to one of the two following study arms:

1. Double dosing (intervention arm) or
2. Standard of care (control arm).

Randomization will be stratified by center. The randomization sequence is generated by a dedicated computer randomization software program (i.e. Castor EDC). Randomization will be performed by the treating physician, coordinating investigator or the local investigator. After randomization each patient will be given a unique patient study number. All data capture will be performed in castor EDC, a program validated and compliant with the General Data Protection Regulation and therefore guaranteeing the privacy of the participants of the study.

#### Study procedures and assessments

**Fig. 1:**
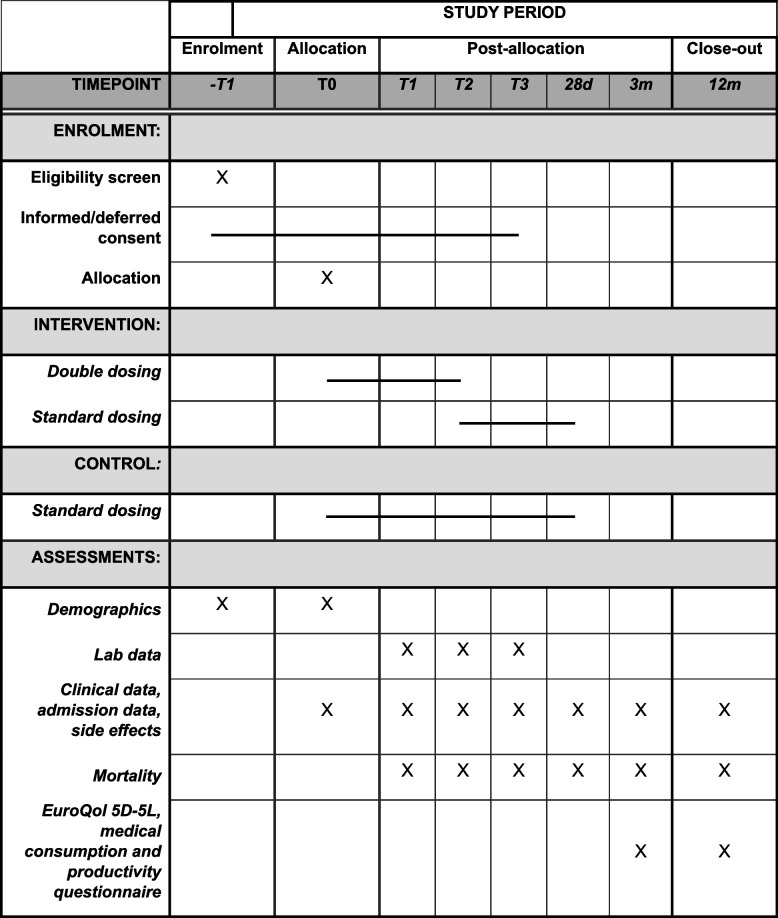
Study assessments, interventions, sample and data collection

An overview of study procedures can be found in Fig. 1. Upon inclusion, participants will be randomized according to procedures described above. Participants randomized to the standard of care arm will receive a loading dose, followed by a daily dose of the target antibiotic, chosen according to local and national guidelines. This can be an intermittent or continuous dosing regimen. Maximum loading and daily dosages can be found in Table 1 and 2. Participants randomized to the double dosing arm will receive a loading dose double the standard, except for ceftriaxone because of its long half-life. If patients already received a loading dose more than 2 h prior to inclusion, a full loading dose will again be administered. In case the starting dose was administered within 2 h prior to inclusion, only the remaining part of the loading dose will be given. This double dosing will be continued for 48 h. In this phase blood levels of the antibiotic are largely dependent on the volume of distribution and only slightly influenced by renal clearance. Therefore, intervention dosages will not be adjusted for kidney function. T1 will be defined as the first morning (8am) the day after admittance to the ICU. To count as T1 the patient has to be admitted (and if applicable received double dosing) for at least 8 h. Consequently, this means T1 will be somewhere between 8 and 32 h after admittance. Every following day will be started at 8am the morning after day 1 and day 2 respectively. This procedure has been chosen taking feasibility in mind, since most blood drawings and morning rounds are done in this time period.

**Table 1:** Maximum dosages control arm

	**Loading dose**^**a**^	**Daily dose**
Cefotaxime	1000 mg	4000 mg
Ceftazidime	1000 mg	3000 mg
Ceftriaxone	2000 mg	2000 mg
Cefuroxime	1500 mg	4500 mg
Meropenem	1000 mg	3000 mg
Flucloxacillin	1000 mg	6000 mg
Amoxicillin	1000 mg	6000 mg
Amoxicillin/clavulanic acid	1000/200 mg	4000/800 mg
Piperacillin/tazobactam	4000/500 mg	16,000/2000 mg

^a^if applicable according to local protocol

**Table 2:** Maximum dosages intervention arm

	**Loading dose**^**a**^	**Daily dose**
Cefotaxime	2000 mg	8000 mg
Ceftazidime	2000 mg	6000 mg
Ceftriaxone	2000 mg	4000 mg
Cefuroxime	3000 mg	9000 mg
Meropenem	2000 mg	6000 mg
Flucloxacillin	2000 mg	12,000 mg
Amoxicillin	2000 mg	12,000 mg
Amoxicillin/clavulanic acid	1000/200 mg + 1000 mg amoxicillin	4000/800 mg + 4000 mg amoxicillin
Piperacillin/tazobactam	8000/1000 mg	32,000/4000 mg

^a^if applicable according to local protocol

Blood samples will be drawn just before antibiotic administration at T1, T2, and T3. They will be kept on ice or in the fridge (2–8 °C) and frozen < 24 h after withdrawal. Samples from participating hospitals, will be transported to the Pharmacy laboratory of the Erasmus MC in bulk and stored at −80 °C or −70 °C until analysis. Plasma concentrations of study antibiotics will be determined by a validated liquid chromatography-mass spectrometry method (LC–MS/MS) [23].

Not all participating centra routinely measure procalcitonin. Because this parameter is indicative of the severity of sepsis and the response to therapy [24], this will be determined in bulk at the Erasmus MC department of Clinical Chemistry.

#### Primary endpoint

The primary endpoint is all cause 28-day mortality as registered in the electronic medical records.

#### Secondary endpoint(s)

Secondary endpoints include 90-day, 365-day, ICU, and hospital mortality as registered in the electronic medical records. As well as ICU and hospital length of stay.

Other clinical parameters include sequential organ failure assessment (SOFA) scores. They will be registered at baseline (T0), 24 (T1), 48 (T2) and 72 (T3) hours, or in any case, after discharge/transfer/death before T3. The SOFA scoring system is used to predict clinical outcomes of critically ill patients. The score is based on six different domains, one each for the respiratory, cardiovascular, hepatic, coagulation, renal and neurological system. In each domain 0–4 points are assigned based on clinical and laboratory findings, resulting in a total score ranging from 0 to 24 points. A higher total score is unfavorable. This scoring system has widely been used since 1996 [25].

Delta (Δ) SOFA, is defined as the score on a fixed time after randomization minus the baseline score. Delta SOFA at T3 is defined as the SOFA at T3 minus SOFA at T0. In case of discharge from the ICU a SOFA of 0 points will be registered. In contrast, in case of death of a participant 24 points will be assigned [26]. Using the delta SOFA allows to compare organ dysfunction at any time point from baseline in the trial arms. Treatment effects on delta SOFA are reliably and consistently associated with mortality in RCTs [26].

Time to shock reversal, defined as the time in hours from inclusion to the moment vasopressors have been administered at a dosage < 0.1 µg/kilogram/minute for at least 4 h, will be determined as well as daily lactate levels and procalcitonin levels.

Clinical cure will be defined as the completion of the β-lactam antibiotic treatment course by day 14 without recommencement of antibiotic therapy within 48 h of cessation for the same infectious episode.

Microbiological eradication will be defined as eradication of the causative organism from the primary source up to 30 days after therapy when confirmed by at least one repeated culture. In cases where there were no repeat cultures and the patient had resolution of the infection, microbial eradication will be presumed.

The pharmacodynamic target will be defined as 100%fT > 4xMIC. Since antibiotic treatment in sepsis will be started empirically, the epidemiological cut-off value (ECOFF) will be used as MIC [27, 28]. The presumed pathogen and matching MIC_ecoff_ are listed in Table 3.

**Table 3:** Presumed microorganism and MIC

**Target antibiotic**	**Presumed Microorganism (S)**	MIC_ECOFF_^a^ (mg/L)
Cefotaxime	Enterobacterales (group)	0.25
Ceftazidime	Pseudomonas aeruginosa	8
Ceftriaxone	Enterobacterales (group)	(0.125)^b^
Cefuroxime	Enterobacterales (group)	8^c^
Amoxicillin	Enterobacterales (group)	8
Amoxicillin/clavulanic Acid	Enterobacterales (group)	8
Flucloxacillin	Staphylococcus aureus	1
Piperacillin/tazobactam	Pseudomonas aeruginosa	16
Meropenem	Pseudomonas aeruginosa	2

^a^European Committee on Antimicrobial Susceptibility Testing. Data from the EUCAST MIC distribution website, last accessed 12–8–2024″. https://www.eucast.org

^b^between brackets in case only a tentative ECOFF is available

^c^The value of 8 mg/L is below the highest ECOFF within the group, but since the clinical breakpoint is also R > 8 mg/L it was decided to keep this value at 8 mg/L

The safety of the intervention will be determined by comparing the number of adverse events (AEs), serious adverse events (SAEs) and suspected unexpected serious adverse reactions (SUSARs).

Health related quality of life (HRQoL) at 3 and 12 months will be assessed using the EuroQoL 5D-5L™ (EQ5D) questionnaire. This questionnaire consists of five questions each representing a dimension of HRQoL. The dimensions are mobility, self-care, usual activities, pain or discomfort and anxiety or depression. Patients can assign a score of no (1), slight (2), moderate (3) or severe problems (4), or are unable to (5) to each of these dimensions. Based on these five dimensions with 5 possible answer levels each, 3,125 health states can be discerned.

Furthermore, an empirical cost-effectiveness analysis will be conducted comparing double dosing to the standard of care following the recommendations of the Dutch guideline for conducting economic evaluations in healthcare (Dutch EE guideline). [29] The time horizon will be equal to the study follow-up period and will assume a healthcare perspective. The latter includes costs for (i) hospital admissions (ICU and other wards), drug or transfusion (ii) acquisition, and (iii) administration, (iv) laboratory diagnostics, and (v) other healthcare resource use such as time spent by health care professionals for consultations, or bedside procedures. Healthcare resource use will be valued with Dutch reference prices from the Dutch EE guideline or taken from our recent costing study [30]. Since differences in costs of informal care time, productivity losses, and travel are irrespective of the chosen strategy and hence not expected, a societal perspective is not assumed. The primary outcome of this cost analysis will be the incremental cost-effectiveness ratio (ICER) per change in mortality of double dosing compared to standard of care. Secondary outcomes of this cost analysis will include total costs per strategy and patient, and the ICER per change in SOFA score and quality-adjusted life year (QALY) gained. All costs will be expressed in Euros and indexed to the reference (to be determined) when necessary.

Subgroup analyses will be performed regarding specific patient groups, type of antibiotic, severity of sepsis, infection site and use of comedication.

### Statistical analysis

In general, *p*-values < 0.05 are considered to indicate statistical significance (2-tailed test). The p-values for the secondary endpoints will be presented but considered descriptive and hypothesis generating rather than confirmatory. Both R studio and Graphpad software will be used for statistical analysis and making graphs, respectively.

All analyses will be performed according to the intention-to-treat (ITT) principle. The ITT population will consist of all patients who have been randomized, irrespective of withdrawals, dropouts or other reasons for failing to complete the study. A per-protocol analysis will be performed as a sensitivity analysis.

#### Baseline characteristics

Descriptive statistics will be used to describe the baseline characteristics. Continuous variables will be described using means (SDs) or medians (interquartile range) depending on the normality of the distribution. Categorical variables will be described using numbers (percentages).

#### Primary endpoint

The primary endpoint, 28-day mortality, will be analyzed using a mixed-effects binary logistic regression [31]. This regression will include treatment effect and source of sepsis as fixed effects and site as random effect. Odds Ratios (OR) and 95% confidence intervals (95% CI) will be reported. Crude proportions by treatment arm will also be reported with an unadjusted OR (95% CI), absolute risk difference (95% CI) and associated p-values.

#### Secondary endpoint(s)

Secondary outcomes are ICU and hospital mortality, 3 months and 1 year mortality, hospital and ICU length of stay, microbiological eradication, time to shock reversal, clinical cure, cost of treatment, quality of life, side effects, Delta PCT (Baseline – Day 3), Delta lactate (Baseline – Day 3), SOFA day 3, Delta SOFA (Baseline – Day 3) and pharmacodynamic target attainment. A similar analysis approach will be taken for the secondary outcomes as for the primary outcome, while for continuous and/or count variables multivariate linear or Poisson regressions will be used, respectively. Missing data, where applicable, will be imputed with the use of multiple imputation under the missing-at-random assumption with chained equations. In the case of missing baseline data, they will be imputed based on baseline characteristics (age, sex, APACHE IV) [32]. The outcome values are not imputed as per convention.

#### Interim analysis

An interim analysis is planned at half of the anticipated sample (*n* = 494 patients). An alpha < 15% estimated power to demonstrate a significant effect at full enrollment (*n* = 988 patients), was defined as non-binding threshold to stop early for futility. Other parameters will be considered as well such as recruitment speed, funding parameters and/or external events that prohibit the completion of the trial.

#### Criteria for termination of the trial

A Data Safety Monitoring Board (DSMB) is installed and will advise the research team on the safety and efficacy of the trial. Reasons to advise to terminate the trial might be:

1. Safety Concerns: If there is a significant increase in adverse events or serious adverse events in the treatment group or if there are any unexpected safety issues that pose risks to participants' health.
2. Ethical Considerations: If new information emerges during the trial that makes the study unethical to continue, such as the emergence of more effective treatment options or other compelling reasons.

### Data monitoring

Because of the nature of the trial with a small chance of slight damage (negligible risk), an independent monitor will visit each study site every 12 months. 10% of all cases will be randomly selected for verification by the independent monitor. Informed consent, source data and reported serious adverse events (SAEs) are reviewed for errors. The data will be pseudonymized when stored in the database and then used for analysis.

### Serious adverse events

SAEs related to known and anticipated side effects of the study antibiotics, pre-existing medical conditions, events with established causality unrelated to the study medication, successfully managed events, and expected laboratory abnormalities will be documented, but not immediately reported. These events will be included in regular safety updates to both the medical ethics committee and the DSMB. All other SAEs and SUSARs will be reported to the local medical ethics committee and DSMB within 7 days of occurrence. Research staff is trained how to address SAEs and how to report these to the coordinating researcher.

## Discussion

The BULLSEYE trial is a randomized controlled study designed to enhance the treatment of critically ill patients with septic shock. The concept of administering higher and double doses of beta-lactams in such patients has got increasing attention over the past few years. To our knowledge, this study is the first prospective trial investigating a short term higher dosing regimen.

Higher dosing naturally comes with an increased risk of toxicity. However, no additional toxicity was observed with increased dosages in the DOLPHIN study [14]. Furthermore, a survey was carried out in our international consortium (including investigators from Belgium, France, and Australia). All collaborators agreed that double dosing during a short period (of 48 h) would lead to improved target attainment and would outbalance the possible risk of toxicity for all antibiotics. Furthermore, a Data Safety Monitoring Board (DSMB) has been established to offer objective advice on the safety and efficacy of the trial during interim analyses and annual meetings. It should be noted however, that in a critically ill patient it is very challenging to differentiate between adverse effects of study medication, other administered medications or the medical condition itself.

Furthermore, inherent to the critically ill population there is a significant heterogeneity in patient, hospital and physician related factors. Standard dosing protocols differ per hospital site, including continuous and intermittent infusions, differences in loading dosage and differences in antibiotic used for selective bowel decontamination. Patient and physician related factors include comorbidity, decisions to continue or discontinue treatment, adherence to sepsis bundles and many more. All of these will influence the results of this study.

One significant challenge anticipated in this trial is the recruitment of patients. This is particularly difficult because inclusion and randomization must be completed promptly after ICU admission. To address this, the inclusion rate will be monitored at multiple stages throughout the trial. If necessary, additional study sites will be recruited to ensure sufficient enrollment and maintain the integrity of the study.

The key strengths of this study include its large sample size, which contributes to a greater comparability between the study arms within this heterogeneous patient population. Importantly, the intervention in this trial is not dependent on patient-specific factors such as age, body mass index, or renal function (including augmented renal clearance). Although a patient-centered approach would be preferable, the strength of this study lies in the timely administration of the antibiotic, necessitating a pragmatic approach.

Overall, the BULLSEYE trial is one of the first trials investigating double dosing in the initial phase of septic shock. Its findings have the potential to significantly impact the future of sepsis treatment in the critically ill, providing a foundation for improved therapeutic strategies and patient outcomes.

## Trial Status

Recruitment began at the first site in January 2025 and is expected to be completed by December 2026.

## Data Availability

The datasets used and/or analyzed during the current trial are available from the corresponding author on reasonable request after publication. The data will need to be requested in the context of research approved by a medical ethical committee and will need to follow the General Data Protection Regulation.

## Abbreviations

AE: Adverse Event
DSMB: Data Safety Monitoring Board
ECOFF: Epidemiological cut-off breakpoints
ECMO: Extracorporeal Membrane Oxygenation
EDC: Electronic Data Capture
HRQoL: Health-related Quality of Life
ICER: Incremental cost-effectiveness ratio
ICU: Intensive care unit
MIC: Minimal inhibitory concentration
PD: Pharmacodynamic
PDT: Pharmacodynamic Target
PK: Pharmacokinetic
(S)AE: (Serious) Adverse Event
SDD: Selective Digestive tract Decontamination
SoC: Standard of Care
SOFA: Sequential Organ Failure Assessment Score
SUSAR: Suspected Unexpected Serious Adverse Reaction
TDM: Therapeutic drug monitoring

## References

[1] van Gestel A, Bakker J, Veraart CP, van Hout BA. Prevalence and incidence of severe sepsis in Dutch intensive care units. Crit Care. 2004;8(4):R153–62. 15312213 10.1186/cc2858 PMC522831

[2] Angus DC, Linde-Zwirble WT, Lidicker J, Clermont G, Carcillo J, Pinsky MR. Epidemiology of severe sepsis in the United States: analysis of incidence, outcome, and associated costs of care. Crit Care Med. 2001;29(7):1303–10. 11445675 10.1097/00003246-200107000-00002

[3] Martin GS, Mannino DM, Eaton S, Moss M. The epidemiology of sepsis in the United States from 1979 through 2000. N Engl J Med. 2003;348(16):1546–54. 12700374 10.1056/NEJMoa022139

[4] Alberti C, Brun-Buisson C, Burchardi H, Martin C, Goodman S, Artigas A, et al. Epidemiology of sepsis and infection in ICU patients from an international multicentre cohort study. Intensive Care Med. 2002;28(2):108–21. 11907653 10.1007/s00134-001-1143-z

[5] Ferrer R, Artigas A, Suarez D, Palencia E, Levy MM, Arenzana A, et al. Effectiveness of treatments for severe sepsis: a prospective, multicenter, observational study. Am J Respir Crit Care Med. 2009;180(9):861–6. 19696442 10.1164/rccm.200812-1912OC

[6] Vincent JL, Sakr Y, Sprung CL, Ranieri VM, Reinhart K, Gerlach H, et al. Sepsis in European intensive care units: results of the SOAP study. Crit Care Med. 2006;34(2):344–53. 16424713 10.1097/01.ccm.0000194725.48928.3a

[7] van der Slikke EC, Beumeler LFE, Holmqvist M, Linder A, Mankowski RT, Bouma HR. Understanding Post-Sepsis Syndrome: How Can Clinicians Help? Infect Drug Resist. 2023;16:6493–511. 37795206 10.2147/IDR.S390947 PMC10546999

[8] Luijks ECN, van der Slikke EC, van Zanten ARH, Ter Maaten JC, Postma MJ, Hilderink HBM, et al. Societal costs of sepsis in the Netherlands. Crit Care. 2024;28(1):29. 38254226 10.1186/s13054-024-04816-3 PMC10802003

[9] Gatti M, Cojutti PG, Pea F. Impact of attaining aggressive vs. conservative PK/PD target on the clinical efficacy of beta-lactams for the treatment of Gram-negative infections in the critically ill patients: a systematic review and meta-analysis. Crit Care. 2024;28(1):123. 10.1186/s13054-024-04911-5 PMC11020314 38627763

[10] Udy AA, Putt MT, Boots RJ, Lipman J. ARC–augmented renal clearance. Curr Pharm Biotechnol. 2011;12(12):2020–9. 21554215 10.2174/138920111798808446

[11] Goncalves-Pereira J, Povoa P. Antibiotics in critically ill patients: a systematic review of the pharmacokinetics of beta-lactams. Crit Care. 2011;15(5):R206. 21914174 10.1186/cc10441 PMC3334750

[12] Abdulla A, Dijkstra A, Hunfeld NGM, Endeman H, Bahmany S, Ewoldt TMJ, et al. Failure of target attainment of beta-lactam antibiotics in critically ill patients and associated risk factors: a two-center prospective study (EXPAT). Crit Care. 2020;24(1):558. 32933574 10.1186/s13054-020-03272-z PMC7493358

[13] Roberts JA, Paul SK, Akova M, Bassetti M, De Waele JJ, Dimopoulos G, et al. DALI: defining antibiotic levels in intensive care unit patients: are current beta-lactam antibiotic doses sufficient for critically ill patients? Clin Infect Dis. 2014;58(8):1072–83. 24429437 10.1093/cid/ciu027

[14] Ewoldt TMJ, Abdulla A, Rietdijk WJR, Muller AE, de Winter BCM, Hunfeld NGM, et al. Model-informed precision dosing of beta-lactam antibiotics and ciprofloxacin in critically ill patients: a multicentre randomised clinical trial. Intensive Care Med. 2022;48(12):1760–71. 36350354 10.1007/s00134-022-06921-9 PMC9645317

[15] Abdulla A, Ewoldt TMJ, Hunfeld NGM, Muller AE, Rietdijk WJR, Polinder S, et al. The effect of therapeutic drug monitoring of beta-lactam and fluoroquinolones on clinical outcome in critically ill patients: the DOLPHIN trial protocol of a multi-centre randomised controlled trial. BMC Infect Dis. 2020;20(1):57. 31952493 10.1186/s12879-020-4781-x PMC6969462

[16] Rietdijk WJR, Drager S, Endeman H, Koch BCP. Beta-lactam Therapeutic Drug Monitoring in Critically ill Patients: Learnings for Future Research. Clin Infect Dis. 2023;77(4):663–4. 37040603 10.1093/cid/ciad215

[17] Ewoldt TMJ, Abdulla A, Rietdijk WJR, Hunfeld N, Muller AE, Endeman H, et al. Which patients benefit from model-informed precision dosing of beta-lactam antibiotics and ciprofloxacin at the ICU? Int J Antimicrob Agents. 2023;62(4): 106931. 37482257 10.1016/j.ijantimicag.2023.106931

[18] Imani S, Buscher H, Day R, Gentili S, Jones GRD, Marriott D, et al. An evaluation of risk factors to predict target concentration non-attainment in critically ill patients prior to empiric beta-lactam therapy. Eur J Clin Microbiol Infect Dis. 2018;37(11):2171–5. 30120647 10.1007/s10096-018-3357-9

[19] Alobaid AS, Brinkmann A, Frey OR, Roehr AC, Luque S, Grau S, et al. What is the effect of obesity on piperacillin and meropenem trough concentrations in critically ill patients? J Antimicrob Chemother. 2016;71(3):696–702. 26702922 10.1093/jac/dkv412

[20] Carrie C, Chadefaux G, Sauvage N, de Courson H, Petit L, Nouette-Gaulain K, et al. Increased beta-Lactams dosing regimens improve clinical outcome in critically ill patients with augmented renal clearance treated for a first episode of hospital or ventilator-acquired pneumonia: a before and after study. Crit Care. 2019;23(1):379. 31775840 10.1186/s13054-019-2621-4 PMC6881978

[21] Shankar-Hari M, Phillips GS, Levy ML, Seymour CW, Liu VX, Deutschman CS, et al. Developing a New Definition and Assessing New Clinical Criteria for Septic Shock: For the Third International Consensus Definitions for Sepsis and Septic Shock (Sepsis-3). JAMA. 2016;315(8):775–87. 26903336 10.1001/jama.2016.0289 PMC4910392

[22] Hernandez G, Ospina-Tascon GA, Damiani LP, Estenssoro E, Dubin A, Hurtado J, et al. Effect of a Resuscitation Strategy Targeting Peripheral Perfusion Status vs Serum Lactate Levels on 28-Day Mortality Among Patients With Septic Shock: The ANDROMEDA-SHOCK Randomized Clinical Trial. JAMA. 2019;321(7):654–64. 30772908 10.1001/jama.2019.0071 PMC6439620

[23] Abdulla A, Bahmany S, Wijma RA, van der Nagel BCH, Koch BCP. Simultaneous determination of nine beta-lactam antibiotics in human plasma by an ultrafast hydrophilic-interaction chromatography-tandem mass spectrometry. J Chromatogr B Analyt Technol Biomed Life Sci. 2017;1060:138–43. 28618388 10.1016/j.jchromb.2017.06.014

[24] de Jong E, van Oers JA, Beishuizen A, Vos P, Vermeijden WJ, Haas LE, et al. Efficacy and safety of procalcitonin guidance in reducing the duration of antibiotic treatment in critically ill patients: a randomised, controlled, open-label trial. Lancet Infect Dis. 2016;16(7):819–27. 26947523 10.1016/S1473-3099(16)00053-0

[25] Vincent JL, Moreno R, Takala J, Willatts S, De Mendonca A, Bruining H, et al. The SOFA (Sepsis-related Organ Failure Assessment) score to describe organ dysfunction/failure. On behalf of the Working Group on Sepsis-Related Problems of the European Society of Intensive Care Medicine. Intensive Care Med. 1996;22(7):707–10. 10.1007/BF01709751 8844239

[26] de Grooth HJ, Geenen IL, Girbes AR, Vincent JL, Parienti JJ, Oudemans-van Straaten HM. SOFA and mortality endpoints in randomized controlled trials: a systematic review and meta-regression analysis. Crit Care. 2017;21(1):38. 28231816 10.1186/s13054-017-1609-1 PMC5324238

[27] Smekal AK, Furebring M, Lipcsey M, Giske CG. Swedish multicentre study of target attainments with beta-lactams in the ICU: which MIC parameter should be used? J Antimicrob Chemother. 2023;78(12):2895–901. 37897332 10.1093/jac/dkad327 PMC10689903

[28] Mouton JW, Muller AE, Canton R, Giske CG, Kahlmeter G, Turnidge J. MIC-based dose adjustment: facts and fables. J Antimicrob Chemother. 2018;73(3):564–8. 29216348 10.1093/jac/dkx427

[29] ZorginstituutNederland. Guideline for conducting economic evaluations in healthcare [in Dutch: richtlijn voor het uitvoeren van economische evaluaties in de gezondheidszorg]. 2024. https://www.zorginstituutnederland.nl/publicaties/publicatie/2024/01/16/richtlijn-voor-het-uitvoeren-van-economische-evaluaties-in-de-gezondheidszorg.

[30] Ewoldt TMJ, Abdulla A, Hunfeld NGM, Muller AE, Gommers D, Polinder S, et al. Health Care Costs of Target Attainment for Beta-Lactam Antibiotics in Critically Ill Patients: A Retrospective Analysis of the EXPAT Study. Ther Drug Monit. 2022;44(1):224–9. 33770020 10.1097/FTD.0000000000000891 PMC8746885

[31] Kahan BC. Accounting for centre-effects in multicentre trials with a binary outcome - when, why, and how? BMC Med Res Methodol. 2014;14:20. 24512175 10.1186/1471-2288-14-20 PMC3923100

[32] Kontopantelis E, White IR, Sperrin M, Buchan I. Outcome-sensitive multiple imputation: a simulation study. BMC Med Res Methodol. 2017;17(1):2. 28068910 10.1186/s12874-016-0281-5 PMC5220613

