# Correction: Standard versus double dosing of beta-lactam antibiotics in critically ill patients with sepsis: The BULLSEYE study protocol for a multicenter randomized controlled trial

**DOI:** 10.1186/s12879-025-10877-8

**Published:** 2025-04-07

**Authors:** M. M. B. Horstink, D. R. Geel, C. A. den Uil, P. E. Deetman, H. Endeman, A. Abdulla, T. M. Bosch, W. J. R. Rietdijk, F. W. Thielen, J. J. Haringman, P. van Vliet, T. A. Rijpstra, C. Bethlehem, A. Beishuizen, A. E. Muller, B. C. P. Koch

**Affiliations:** 1 Department of Intensive Care, Maasstad Hospital, Rotterdam, The Netherlands; 2 Rotterdam Clinical Pharmacometrics Group, Erasmus MC, Rotterdam, The Netherlands; 3 Department of Hospital Pharmacy, Erasmus MC, Rotterdam, the Netherlands; 4 Department of Intensive Care, Albert Schweitzer Hospital, Dordrecht, The Netherlands; 5 Department of Intensive Care, OLVG, Amsterdam, The Netherlands; 6 Department of Intensive Care, Erasmus MC, Rotterdam, The Netherlands; 7 Department of Clinical Pharmacology & Toxicology Maasstadlab, Maasstad Hospital, Rotterdam, The Netherlands; 8 Department of Hospital Pharmacy, Maasstad Hospital, Rotterdam, The Netherlands; 9 School of Health Policy & Management, Erasmus University, Erasmus Centre for Health Economics Rotterdam, Rotterdam, The Netherlands; 10 Department of Intensive Care, Isala Hospital, Zwolle, The Netherlands; 11 Department of Intensive Care Haaglanden Medical Center, The Hague, The Netherlands; 12 Department of Intensive Care, Amphia, Breda, The Netherlands; 13 Department of Intensive Care, Frisius MC, Leeuwarden, The Netherlands; 14 Department of Intensive Care, Medisch Spectrum Twente, Enschede, The Netherlands; 15 Department of Medical Microbiology, Haaglanden Medical Center, The Hague, The Netherlands

**Correction** **:** **BMC Infect Dis 25, 392 (2025)**

**https://doi.org/10.1186/s12879-025-10747-3**

Following publication of the original article [1], we were notified that the list of beta-lactam antibiotics was incorrectly written in the Methods section of the Abstract as follows: cefuroxime, ceftazidime, ceftriaxone, cefotaxime, amoxicillin, amoxicillin/clavulanic acid, flucloxacillin, meropenem, and **piperacillin/clavulanic acid**.

The correct list should be: cefuroxime, ceftazidime, ceftriaxone, cefotaxime, amoxicillin, amoxicillin/clavulanic acid, flucloxacillin, meropenem, and **piperacillin/tazobactam**.

The original article has been corrected.
